# Cognitive symptoms in schizophrenia: an analysis of awareness, assessment, and management practices among psychiatrists and primary care physicians

**DOI:** 10.3389/fpsyt.2025.1567410

**Published:** 2025-05-08

**Authors:** Luis Agüera-Ortiz, Enric Aragonés, Bárbara Buch-Vicente, Juan Manuel Mendive, Mercedes Peña, Eduard Vieta

**Affiliations:** ^1^ Department of Psychiatry, Instituto de Investigación Sanitaria (imas12), Hospital Universitario 12 de Octubre, Madrid, Spain; ^2^ Mental Health Network Research Centre, CIBERSAM, Carlos III Health Institute, Madrid, Spain; ^3^ Constantí Primary Care Centre, Catalan Health Institute, Constantí, Spain; ^4^ Primary Care Research Institute IDIAP Jordi Gol, Barcelona, Spain; ^5^ Department of Basic Psychology, Psychobiology, and Methodology of Behavioral Sciences, Faculty of Psychology, University of Salamanca, Salamanca, Spain; ^6^ Instituto de Investigación Biomédica de Salamanca (IBSAL), Salamanca, Spain; ^7^ La Mina Primary Health Care Academic Centre, Catalan Health Institute, University of Barcelona, Barcelona, Spain; ^8^ Department of Psychiatry, University Hospital Gregorio Marañón, Madrid, Spain; ^9^ Psychiatry and Psychology Department, Hospital Clínic, Institute of Neurosciences, University of Barcelona, IDIBAPS, CIBERSAM, Barcelona, Spain

**Keywords:** schizophrenia, cognitive symptoms, cognitive disorder, screening, diagnosis, awareness, therapeutics

## Abstract

**Introduction:**

Cognitive symptoms contribute to the worsening of functionality in people with schizophrenia. The objective of this study was to explore the current knowledge about cognitive symptoms (relevance, evaluation, and management) of psychiatrists and primary care physicians (PCPs) involved in the care of patients with schizophrenia in Spain.

**Methods:**

The study was developed in two phases: a quantitative phase and a qualitative one. Both took place between November 2023 and January 2024. For the quantitative phase, an online questionnaire was developed and administered to 100 psychiatrists and 125 PCPs. In addition, further qualitative data were collected through individual semi-structured telephone interviews. Descriptive analyses and qualitative analyses (induction-deduction approach) were carried out.

**Results:**

Health professionals agreed that cognitive symptoms are present in patients with schizophrenia, with 75% of psychiatrists and 45% of PCPs acknowledging this. Both groups also considered the detection of these symptoms as crucial for improving patient functionality (89% psychiatrists vs 88% PCPs). However, over half of both psychiatrists and PCPs do not consistently evaluate cognitive symptoms, attributing this to factors such as time constraints, limited access to both pharmacological and non-pharmacological treatments, and a lack of effective diagnostic tools. PCPs additionally highlighted insufficient training regarding cognitive symptoms in schizophrenia. Both groups underscored the need for specific treatments for cognitive symptoms, with 87% agreement.

**Conclusion:**

This study offers an overview of the current understanding regarding the relevance, evaluation, and management of various cognitive symptoms according to clinical practice in Spain. The results highlight the necessity for enhanced guidelines, training, and improved access to effective treatments to address cognitive symptoms in patients with schizophrenia.

## 1 Introduction

Schizophrenia is a severe, complex, and chronic mental disorder that affects approximately 24 million people worldwide, equivalent to 1 in 300 people (1). In Spain, prevalence rates show considerable variability, ranging from approximately 0.2% to 0.7% (2–4). Schizophrenia is characterized by the presence of positive, negative and cognitive symptoms (5). Positive symptoms include, among others, hallucinations and delusions; negative symptoms often include apathy, anhedonia, and affective flattening, while cognitive symptoms encompass attention deficits and impaired executive function (6).

Cognitive symptoms are present to varying degrees in 75 to 85% of people with schizophrenia (7), and they typically, show a limited response to antipsychotic treatment (8). Cognitive impairment is a core symptom of schizophrenia, often evident even in the early stages of the illness (9). These deficits tend to appear early in the course of the disease, often preceding the onset of psychotic symptoms (10). The course of cognitive symptoms in schizophrenia can be understood through both neurodevelopmental and neurodegenerative perspectives. Neurodevelopmental abnormalities, such as disrupted brain maturation processes, contribute to cognitive deficits that are evident from childhood or adolescence. These deficits may remain stable or worsen over time, reflecting a developmental lag (11). On the other hand, neurodegenerative processes, including progressive brain changes and loss of neural integrity, can lead to further cognitive decline during adulthood (12). This interplay between neurodevelopmental and neurodegenerative processes underscores the complexity of cognitive impairment in schizophrenia. This contrasts with affective disorders, where cognitive impairments are often linked to mood episodes, and with Alzheimer’s disease, which is characterized by a more uniform and progressive decline in cognitive function due to neurodegeneration (12–15).

Cognitive impairments in schizophrenia are widespread, affecting multiple cognitive domains, including processing speed, verbal memory, executive function, social cognition, and verbal fluency (9, 16). Importantly, cognitive symptoms have a profound impact on patients’ ability to perform daily living activities, leading to deteriorated functional outcomes and higher indirect costs associated with the disease (12, 17–20). In Spain, as in many other countries, schizophrenia represents a significant public health concern, with substantial socioeconomic implications (21).

Currently, there is a lack of consensus on the most effective methods for assessing cognitive symptoms in people with schizophrenia in clinical practice. Some experts advocate for performance-based measurements, while others argue that these measurements are impractical due to their cost and time requirements (22, 23). Interview-based measurements of cognitive and functional change are viewed as more practical, but they validity is limited without informant involvement or frequent contact from clinicians (24). Moreover, there is ongoing research into potential biomarkers that could be additionally used to assess cognitive function in schizophrenia, such as brain-derived neurotrophic factor (BDNF) (25), macrophage migration inhibitory factor (26) and homocysteine (27).

The Schizophrenia Cognition Rating Scale (SCoRS), Brief Assessment of Cognition in Schizophrenia (BACS) (28, 29), and Screen for Cognitive Impairment in Psychiatry (SCIP) (30) are valuable tools for detecting and/or assessing cognitive deficits in schizophrenia. The European Psychiatric Association guidance emphasizes the importance of using structured interview-based tools like SCoRS for assessing cognitive impairment in schizophrenia. SCoRS is mainly used in clinical trials and involves both patient and informant ratings, providing a comprehensive view of cognitive functioning and its impact on daily life. The BACS, designed to assess, in the context of a clinical trial, cognitive domains such as verbal memory, working memory, executive function, motor speed, attention and verbal fluency, is both time-efficient and cost-effective. It is characterized for his brief administration and scoring time (about 30 minutes), portability, repeatability, and availability of alternate forms (28). SCIP is a brief, well-validated screening tool designed to detect cognitive impairments in psychiatric patients, particularly those with psychotic and affective disorders. It consists of five subscales assessing verbal learning, working memory, verbal fluency, delayed recall, and processing speed, and takes about 15 minutes to complete (31). The guidance suggests incorporating SCIP into regular assessments to identify cognitive deficits early and tailor interventions accordingly (22, 23). These assessments provide significant prognostic value, as cognitive deficits are strong predictors of functional outcomes in schizophrenia. By identifying specific cognitive impairments, clinicians can develop personalized treatment plans, including cognitive remediation therapy and pharmacological interventions, to improve patient outcomes. The integration of these tools into routine clinical practice enhances the accuracy of cognitive assessments and informs targeted therapeutic strategies, ultimately contributing to better management of cognitive symptoms in schizophrenia.

Because of this, it is crucial not only to deepen our understanding of the cognitive symptoms associated with schizophrenia but also to analyze how they are evaluated and managed in daily clinical practice. Although scientific evidence highlights the relevance of these cognitive alterations in the functional prognosis of patients (9, 23), it is essential to assess the extent of knowledge that that psychiatrists and primary care physicians (PCPs) have about these symptoms, as well as the importance they place on them in their clinical decision-making and therapeutic approach. Understanding how cognitive symptoms are addressed in clinical practice and whether health professionals implement specific actions for their treatment is essential. Therefore, this study aims to assess the knowledge of psychiatrists and PCPs about the cognitive symptoms associated with schizophrenia, as well as to explore how these symptoms are evaluated and managed in routine clinical practice. Given the significant impact of cognitive problems on patient outcomes, this research may contribute to enhancing the quality of care and functional outcomes for patients with schizophrenia in the Spanish healthcare system.

## 2 Materials and methods

### 2.1 Study design

The study was conducted in two phases: quantitative phase and qualitative phase, both conducted between November 2023 and January 2024. The study design, including the development of the questionnaire and interview guide, and participant selection were validated by a scientific committee composed of seven experts (2 PCPs, 3 psychiatrists, 1 mental health nurse, and 1 neuropsychologist).

The inductive phase involved identifying themes and patterns directly from the data without preconceived categories. In our study, we performed a literature review and analyzed the scientific committee meeting and first interviews transcripts to identify recurring themes related to the awareness, assessment, and management of cognitive symptoms in schizophrenia. This involved coding the transcripts and grouping similar responses to form broader categories (e.g., psychiatrists vs. PCPs) and quantitative answers (e.g. “it can improve the patient’s functionality vs it can improve the patient’s quality of life).

Afterwards, the deductive phase involved comparing the emerging themes from the inductive analysis with established concepts and theories in the field. In our study, we assessed how well the identified themes aligned with known barriers, assessment methods, and management practices documented in the literature. The categorization of interviews by type of participant (psychiatrists vs. PCPs) allowed for a comparison of their perspectives with theoretical expectations about their roles and challenges in managing cognitive symptoms.

### 2.2 Study population

A sample of 100 psychiatrists and 125 PCPs was selected from the IQVIA OneKey database, which provides accurate and real-time information about healthcare professionals, including psychiatrists and PCPs involved in treatment of patients diagnosed with schizophrenia (32).

To calculate the sample size, a sampling error margin of ±8.2% for psychiatrists and ±8.1% for PCPs was established, corresponding to a 95% confidence interval (95% CI), assuming p=q=50%. Samples with a margin of error less than 10% within the 95% CI were deemed representative. The sample set was selected proportionally to the gender of the participants and the different geographical areas of Spain, with all regions being represented.

All participants received compensation commensurate with the fair market value for participating in the study. None of the participants were provided with information about the sponsor of this article or the investigational drugs involved.

### 2.3 Materials

- Online questionnaire: A Computer-Assisted Web Interviewing (CAWI) questionnaire was designed with 53 questions and an estimated duration of 20 minutes. The development of the questionnaire was based on a thorough review of the existing literature on cognitive symptoms associated with schizophrenia and was subsequently validated by the scientific committee. The questionnaire focused on five thematic areas: definition of cognitive symptoms in patients with schizophrenia, epidemiology, disease burden, patient flow, and unmet needs. A Likert scale ranging from 1 to 5 was used for response options, with 1 being of minimal importance and 5 representing maximum importance.

- Semi-structured individual phone interview: A semi-structured interview guide was elaborated, allowing a certain degree of flexibility according to the purposes of the study. The interview guide was aligned with the questions of the quantitative phase, specifically focusing on the understanding of the detection of cognitive symptoms and the follow-up of these patients according to clinical practice in Spain.

### 2.4 Procedures

#### 2.4.1 Quantitative phase

The quantitative phase was carried out through the online questionnaire targeted at 100 psychiatrists and 125 PCPs from different primary care centers and hospitals in Spain. Every healthcare professional could access to the questionnaire using their personal computer. To facilitate this, a link was distributed to the professionals for access. Data gathering for this phase spanned from November 27, 2023, to December 28, 2023.

#### 2.4.2 Qualitative phase

The qualitative phase included semi-structured individual phone interviews with 11 proposed psychiatrists and PCPs, who successfully completed the semi-structured interview. The interviewees were selected to ensure regional representation, prioritizing those who visited more patients with schizophrenia. Six were psychiatrists (2 from mental health centers, 2 from private hospitals and 2 from public hospitals) and five PCPs, from five different regions of Spain (Andalusia, Catalonia, Galicia, Community of Madrid, and Valencian Community).

These interviews were conducted between November 21, 2023, and January 12, 2024. Qualitative interviews were conducted individually by phone with an approximate duration of 1 hour. The interviews were conducted by 2 consultants equivalent trained in the methodology of qualitative interviews. All interviews were recorded for further analysis. Participants’ responses were recorded in an anonymized database.

Qualitative analyses do not pursue sample size representativity but aim to explore complex and specific topics, often with little or no official data available. They rely on direct information from stakeholders. This initial approach helps to refine and focus quantitative analyses, which require statistical representativity, making them more cost-efficient.

### 2.5 Statistical analysis

A descriptive analysis of the data was carried out. Continuous variables were described with the mean, standard deviation (SD), and median. Categorical variables were described as frequencies and percentages. In questions based on scales from 1 to 5, the mean score for each attribute and the sum of the percentage result of scores 4 and 5, called “top 2 boxes” (T2B), were calculated. The significance of the differences between the scores of the psychiatrists and PCPs was also estimated.

For the qualitative interviews, a method of qualitative content analysis with an inductive-deductive approach was used. Once the key and specific questions (sub-themes) were recognized, the aggregated results were analyzed, and illustrative quotes from the interviews were selected to exemplify these themes. Results were stratified by healthcare professional type, specifically distinguishing between psychiatrists and PCPs. This stratification facilitated clearer comparison of responses and practices between these two groups in managing cognitive symptoms in patients with schizophrenia.

## 3 Results

### 3.1 Quantitative phase

#### 3.1.1 Participant demographics and characteristics

A total of 225 healthcare professionals were included in the quantitative phase: 100 psychiatrists (44.4%) and 125 PCPs (55.6%). Of the 225 participants, 100% responded to the questionnaire, and there was a balanced gender distribution between them (50.2% men and 49.8% women). More information regarding sample characteristics can be found in **Supplementary Table 1**.

#### 3.1.2 Recognition of cognitive symptoms

The psychiatrists reported recognizing positive symptoms in 95.0% of their regularly seen schizophrenia patients, negative symptoms in 85.0%, and cognitive symptoms in 73.0%. Conversely, the PCPs identified positive symptoms in 89.6% of schizophrenia patients, negative symptoms in 50.4%, and cognitive symptoms in 44.8%. According to 85.0% of the psychiatrists and 69.6% of the PCPs, the cognitive symptoms in patients with schizophrenia exhibit distinguishing features compared to other disorders that involve cognitive impairment, such as Alzheimer’s disease. The differential aspects are presented in the **Table 1**.

**Table 1:** Differential aspects of cognitive symptoms present in people with schizophrenia by specialty.

Aspects	Psychiatrists (N=100)	PCPs (N=125)	Total (N=225)
It is an inherent deterioration to the pathology, n (%)	47 (55.3%)	42 (48.3%)	89 (51.7%)
It is part of the clinical course of schizophrenia, n (%)	64 (75.3%)	53 (60.9%)	117 (68%)
The repercussions that cognitive symptoms have on the lives of patients with schizophrenia, n (%)	62 (72.9%)	63 (72.4%)	125 (72.7%)
It is more subtle than the cognitive impairment of Alzheimer’s, n (%)	25 (29.4%)	20 (23.0%)	45 (26.2%)
It is more diffuse than in brain lesions, n (%)	29 (34.1%)	26 (29.9%)	55 (32%)
It is more severe than the cognitive impairment of bipolar disorder, n (%)	59 (69.4%)	22 (25.3%)	81 (47.1%)
It is more severe than the cognitive impairment of depression, n (%)	52 (61.2%)	37 (42.5%)	89 (51.7%)
It is not associated with the patient’s age when the evaluation is mad, n (%)	21 (24.7%)	21 (24.1%)	42 (24.4%)
It is not associated with the age of onset of schizophrenia, n (%)	8 (9.4%)	11 (12.6%)	19 (11.1%)
I have no opinion/does not apply to me, n (%)	1 (1.2%)	0 (0.0%)	1 (0.6%)

#### 3.1.3 Importance of cognitive symptom detection

**Figure 1:**
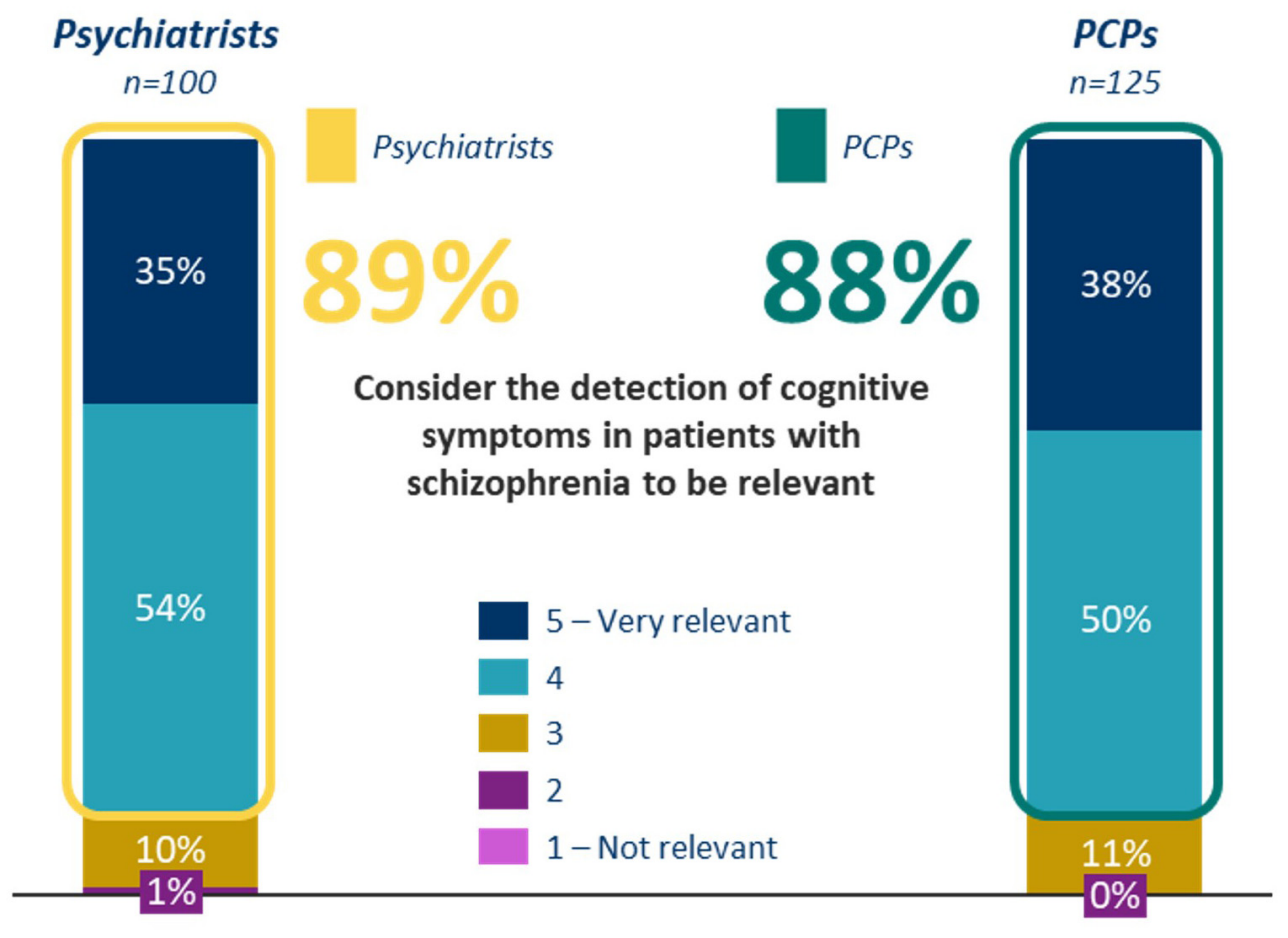
Grade of relevance perceived by psychiatrist and PCPs regarding cognitive symptoms detection.

**Figure 2:**
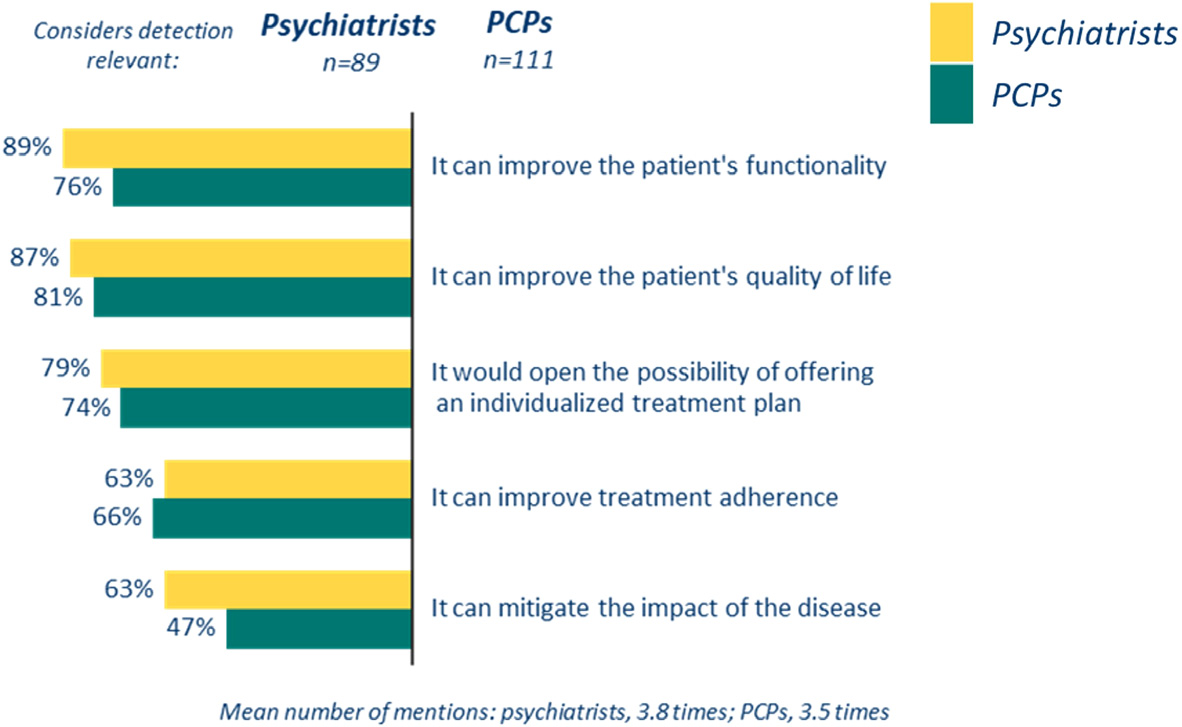
Reasons to detect cognitive symptoms associated with schizophrenia expressed by the psychiatrist and PCPs that considered de detection to be very relevant.

Using a scale of 1 to 5, where 5 represents the highest level of importance, 89.0% of psychiatrists and 88.8% PCPs rated the identification of cognitive symptoms in schizophrenia as a 4 or higher. The main reasons cited for this emphasis included: 1) the potential to improve patient functionality and quality of life, and 2) the opportunity to develop personalized treatment plans (**Figures 1**, **2**).

#### 3.1.4 Assessment methods for cognitive symptoms

In the assessment of cognitive symptoms in patients with schizophrenia, psychiatrists and PCPs reported that they primarily relying on clinical criteria (87.0% and 61.6% respectively) and patient interviews (85.0% and 78.4% respectively). Additionally, 45.0% of psychiatrists and 52.0% of PCPs reported the use of assessment tools, such as the Mini-mental test or the Schizophrenia Cognition Rating Scale (SCoRS) (29, 33). The specific questionnaires or scales used are listed in the **Supplementary Table 2**. Furthermore, 65.0% of psychiatrists and 75.2% of PCPs stated that they always or very frequently record cognitive symptoms associated to schizophrenia in the patient’s medical record.

#### 3.1.5 Recording and follow-up of cognitive symptoms

During the follow-up of patients presenting cognitive symptoms, 52.0% of psychiatrists and 49.6% of PCPs noted that they do not systematically evaluate these symptoms. For psychiatrists, the primary reason is the limited access to non-pharmacological treatments, such as cognitive rehabilitation (69.2%), while for PCPs the main cause was the lack of awareness and training about cognitive symptoms in schizophrenia (75.8%) (**Table 2**).

**Table 2:** Reasons for not systematically assessing cognitive symptoms during follow-up visits by specialty.

Reason	Psychiatrists (N=52)	PCPs (N=62)	Total (N=114)
It is not a relevant aspect and I consider it within the negative symptoms of schizophrenia	2 (3.8%)	2 (3.2%)	4 (3.5%)
I do not know which professional I should refer to	1 (1.9%)	1 (1.6%)	2 (1.8%)
Lack of awareness/training about cognitive symptoms in schizophrenia	29 (55.8%)	47 (75.8%)	76 (66.7%)
Lack of time	35 (67.3%)	41 (66.1%)	76 (66.7%)
Lack of diagnostic and/or evaluation tools	32 (61.5%)	35 (56.5%)	67 (58.8%)
Lack of pharmacological treatments	24 (46.2%)	13 (21%)	37 (32.5%)
Lack of non-pharmacological treatments such as, for example, availability of cognitive rehabilitation	36 (69.2%)	17 (27.4%)	53 (46.5%)
Others	0 (0.0%)	4 (6.5%)	4 (3.5%)

#### 3.1.6 Impact of cognitive symptoms on daily life

**Figure 3:**
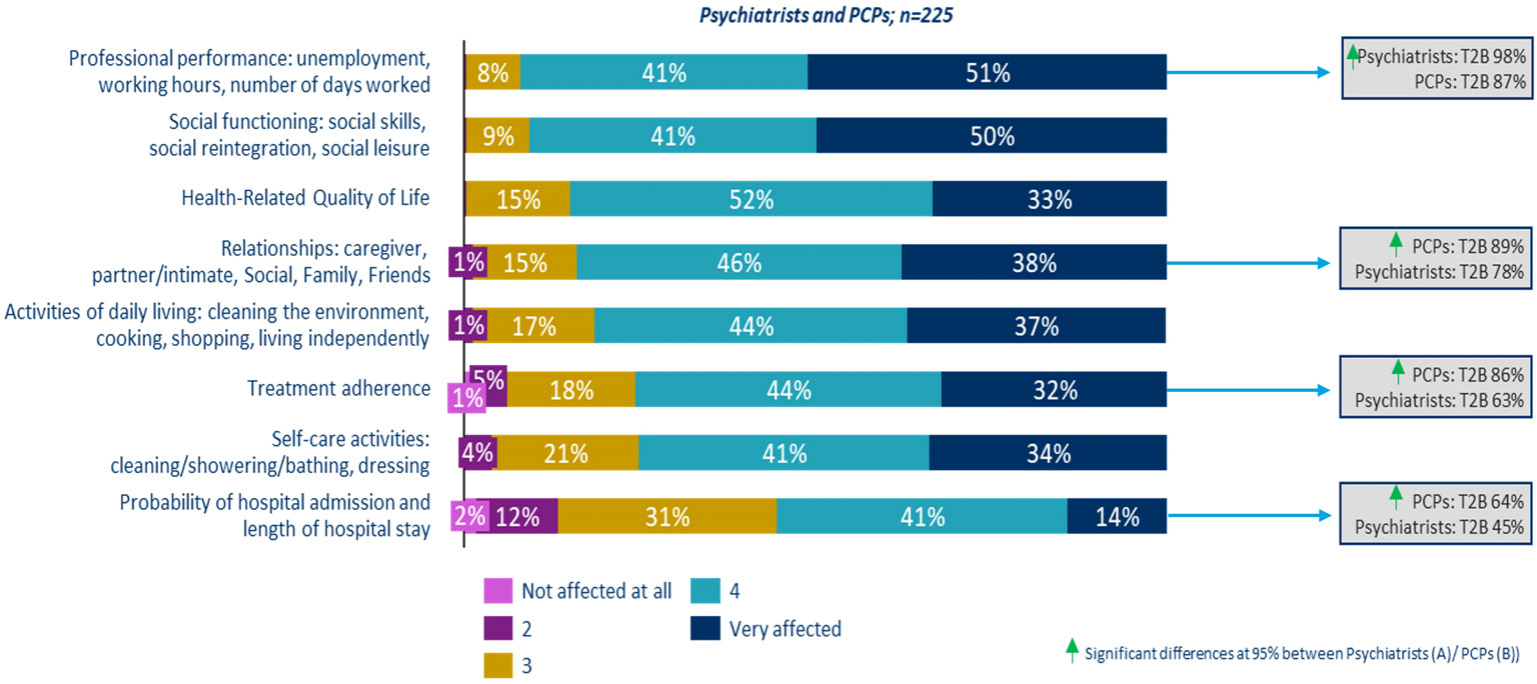
Aspects of daily life that are affected in a patient with cognitive symptoms associated with schizophrenia.

Furthermore, when specifically asked about their knowledge and application of assessment scales for monitoring cognitive symptoms, only 10.0% of psychiatrists and 19.2% of PCPs reported knowing and using such tools. Based on the average responses of psychiatrists and PCPs (calculated using T2B methodology), the daily life aspects most affected in patients with schizophrenia include 1) professional performance, which encompasses unemployment, working hours, and number of days worked (93.0%); 2) social functionality, defined as the development of social skills, reintegration, and leisure (90.7%), and 3) health-related quality of life (84.9%) (**Figure 3**) (34).

Despite indicating a high impact on most areas of a patient’s daily life, significant differences were observed between psychiatrists and PCPs in certain aspects. Psychiatrists were more likely to perceive that professional performance is severely impacted (98.0% vs. 87.2%), whereas PCPs reported that patients’ close relationships (88.8% vs. 78.0%), treatment adherence (85.6% vs. 63.0%), and the likelihood and duration of hospitalization (64.0% vs. 45.0%) are more adversely affected (**Figure 3**).

#### 3.1.7 Unmet needs in cognitive symptom management

**Figure 4:**
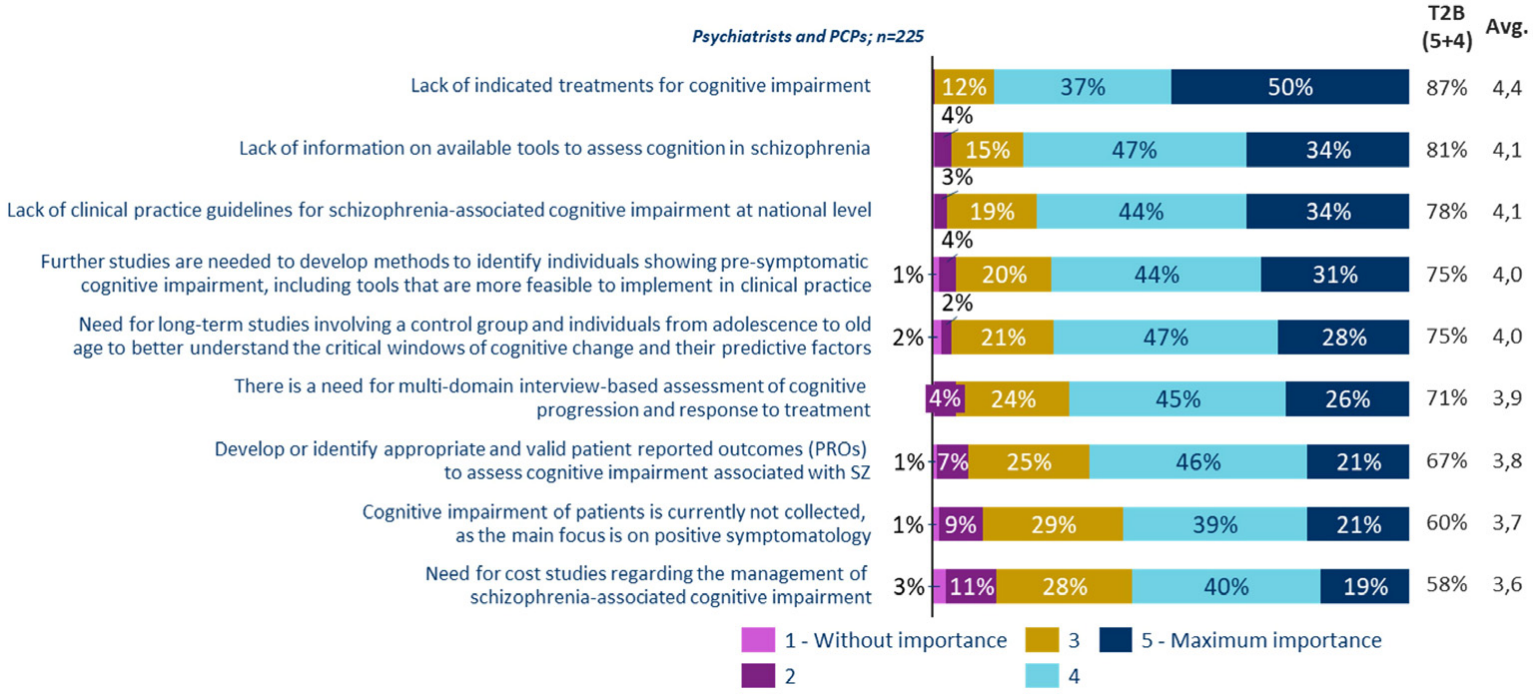
Unmet needs in the management of patients with cognitive symptoms associated with schizophrenia.

Regarding unmet needs in the management of patients with cognitive symptoms associated with schizophrenia, nearly all psychiatrists and PCPs punctuated with high scores the importance of having specific treatments for these cognitive symptoms. On a scale of 1 to 5, 87.5% of psychiatrists and PCPs rated the necessity of treatments for cognitive symptoms at 4 or higher. Additionally, 80.9% highlighted the limited information regarding the tools available for evaluating cognition in schizophrenia. Moreover, 78.2% pointed out the absence of national clinical practice guidelines specifically aimed at managing cognitive symptoms in schizophrenia (**Figure 4**).

### 3.2 Qualitative phase

During the qualitative interviews, psychiatrists claimed to play a crucial role in the overall monitoring of patients, particularly within the mental health center where most patients are referred for treatment and follow-up. They identified themselves as the primary point of contact for patients, actively involved in their holistic care and primarily responsible for the detection of cognitive symptoms. They described themselves as the key decision-makers regarding treatment and were deeply engaged in managing cognitive symptoms (**Table 3**). Psychiatrists advocated for pharmacological treatment targeting cognitive symptoms in people with schizophrenia.

**Table 3:** Role of the psychiatrist and the PCP in the detection of cognitive symptoms according to qualitative interviews.

Topic	Interviewee’s Specialty	Quote
Role of the psychiatrist and the PCP	Psychiatrist	The role of the PCP depends on the level of involvement of each doctor, but in general it is quite limited. These patients are under our care, that is, the primary care doctors mainly focus on renewing prescriptions when necessary. The function often boils down to conveying information from families; for example, a mother might say that she perceives certain symptoms. It’s more like a consultation
	PCP	From Primary Care, the treatment could be changed, but it’s not the case. The psychiatrist is the one who manages these patients. We do not intervene in the antipsychotic treatment”; “Stable patients usually come when they realize they are getting worse; they realize it. I don’t schedule appointments for those who are well controlled unless they need something or for medication renewal
Detection of cognitive symptoms	Psychiatrist	The detection of cognitive symptoms is done exclusively through clinical examination. The use of questionnaires is more reserved for research tasks; it’s more about the general clinical impression when the patient tells us they have problems
	PCP	Differentiating cognitive symptoms from negative symptoms is a challenge, it would require a deeper evaluation. This is usually done more by psychiatrists. If the patient cooperates, they can be distinguished based on the information provided by their caregiver or their closest relative, you can conduct interviews and distinguish between symptoms. However, it is a complicated task”; “Specific scales are not used to monitor the cognition of these patients. Their progress is observed and followed based on the patient’s knowledge and how they see their evolution
Annotation in the Clinical Record of cognitive symptoms	Psychiatrist	The symptoms are simply noted: psychotic contact, social impoverishment, unspecified cognitive deterioration, and problems of this kind. They report memory losses, among other things. However, it is not something highly standardized or strictly regulated
	PCP	A description of the observed symptoms related to cognitive impairment is recorded in the patient’s medical history, with the intention of taking them into account and tracking the progression in subsequent visits

**Table 3** outlines the general roles of the psychiatrist and PCP, as well as their specific responsibilities in detecting and documenting cognitive symptoms. The PCPs claimed that they are responsible for the overall monitoring of patient health. During routine follow-up visits of patients, their work includes detecting early symptoms or unusual behaviors and supporting the psychiatrist in monitoring the treatment and health of these patients. Most of the interviewed PCPs admitted to having limited awareness of cognitive symptoms in patients with schizophrenia (**Table 3**). Importantly, both psychiatrists and PCPs reported that they only use scales and questionnaires solely in a research context, rather than in their everyday clinical practice (**Table 3**).

## 4 Discussion

Current assessment and treatment options for cognitive impairment in schizophrenia involve comprehensive evaluations of neurocognitive and social cognitive domains using standardized tools, observer reports, and self-reports. Despite these methods, cognitive functioning is still poorly assessed in clinical practice.

Previous guidelines from the European Psychiatric Association stress the importance of systematic cognitive assessments and offer recommendations for optimal evaluation (22, 23). However, these guidelines have not been fully implemented, influenced probably by the lack of a specific, updated and national guideline, indicating a need for further dissemination and application to improve patient care and outcomes.

The results of this study indicate that the significant majority of psychiatrists and PCPs recognize cognitive symptoms in patients with schizophrenia as distinct features of this condition. They also noted that cognitive impairment is perceived as more severe compared to that experienced by patients with depression and bipolar disorder, data that are aligned with previous published studies (16, 35, 36).

### 4.1 Assessment practices and tools

In detecting cognitive symptoms associated with schizophrenia, clinicians primarily rely on clinical criteria and patient interviews, which may lead to variability in findings across different professionals. Some clinicians reported using standardized scales to aid in the diagnostic process, with the Mini-Mental State Examination (MMSE) (29) and other screening test being the most employed questionnaires (87.3%). However, it is relevant to acknowledge that the MMSE is a general cognitive screening tool that has not been specifically validated for detecting cognitive impairment in schizophrenia. Furthermore, screening tests are designed exclusively for initial assessment; therefore, a thorough neuropsychological evaluation carried out by qualified professionals is essential for validating any diagnostic concerns regarding cognitive dysfunction.

The Measurement and Treatment Research to Improve Cognition in Schizophrenia (MATRICS) initiative (22), recommends a thorough evaluation of the six cognitive domains affected in individuals with schizophrenia (processing speed, attention/vigilance, working memory, verbal learning and memory, visuospatial learning and memory, and reasoning and problem-solving) conducted across all phases of the disorder, as well as in individuals at clinical high risk of psychosis. Furthermore, they also recommended that social cognition be incorporated into these assessments (37), as it is considered an important factor as well (38, 39).

### 4.2 Challenges in routine clinical practice

In this study, professionals reported using scales like the MATRICS Consensus Cognitive Battery (MCCB) (18.2%) (37, 40, 41) and Schizophrenia Cognition Rating Scale (SCoRS) (48.2%), both developed through the MATRICS initiative, especially for use in clinical trials. The MCCB was designed to meet rigorous criteria including the assessment of social cognition and is regarded as the gold standard in performance scales for cognitive evaluation in schizophrenia clinical trials. Despite their strong reliability and validity in assessing cognitive deficits in schizophrenia, the MCCB is typically used only in research contexts due to the specialized training it requires and the lengthy administration time (60–90 min). The SCORS has shown a strong correlation with patients’ functionality (42). However, this scale, while requiring less time, also has its limitations, as its validity depends on the patient’s and caregiver’s awareness of cognitive deficits (22, 43). In the clinical context, recommended alternatives such as the Brief Assessment of Cognition in Schizophrenia (BACS), validated in validated in Spanish (44), and the Screen for Cognitive Impairment in Psychiatry (SCIP), were not frequently utilized by the clinicians in this study (21.8% vs 13.6%). Our results, therefore, highlight the lack of specific training on the multidisciplinary approach to cognitive symptoms associated with schizophrenia, both for diagnosis and for patient follow-up, in clinical settings.

Moreover, qualitative interviews revealed that clinicians primarily use these scales only in the context of research, rather than in clinical practice. Previous studies have shown that formal evaluations of cognitive function in patients with schizophrenia are rarely conducted in clinical settings, mainly due to time constraints during patient visits, limited available tools for screening cognitive symptoms, and the lack of effective and/or specific pharmacological treatments for cognitive symptoms (45, 46). Consequently, the present study reveals that the current process for detecting cognitive symptoms associated with schizophrenia is currently suboptimal and limited.

This situation may be related to the absence of clinical practice guidelines, in the Spanish context, that support the management of such patients. Currently, there are a few guidelines and consensus documents available to guide different professionals in appropriately addressing these cognitive symptoms in patients with schizophrenia (22, 23). Therefore, there is a pressing need to develop specific frameworks and tools to ensure adequate care for these patients.

### 4.3 Impact on patient functionality and quality of life

Despite the absence of specific strategies, it is noteworthy that the majority of professionals emphasized the significance of assessing cognitive symptoms (**Figure 2**). Cognitive deficits are significant predictors of functional outcomes (47), limiting both rehabilitation efforts and treatment throughout the course of this disease (22).Thus, addressing these deficits is particularly relevant for enhancing the functionality and quality of life of patients with schizophrenia. These findings are consistent with previous studies indicating that cognitive symptoms profoundly influence patients’ daily lives, especially concerning occupational and social functioning (12, 21).

Psychiatrists and PCPs assert that identifying cognitive symptoms linked to schizophrenia is essential for improving the most impacted areas: occupational and social functionality. Indeed, research has shown that cognitive impairment serves as a better predictor of employment and social function in schizophrenia than positive and negative symptoms (48), with enhancements in verbal working memory emerging as particularly predictive of employment outcomes (48). This would subsequently enhance overall functionality and lead to a better quality of life. Expert considered to have treatments specifically indicated for cognitive symptoms as the most urgent unmet need.

### 4.4 Economic and healthcare system implications

Furthermore, many professionals believe that the cognitive symptoms associated with schizophrenia considerably impact not only the patients’ daily lives but also the economic and healthcare system. In terms of disease burden, schizophrenia is linked to high rates of unemployment, with 80%-90% of patients with schizophrenia being unemployed and remaining so in adulthood (12, 21, 49). This disease is also associated with lower wages, reduced work hours, decreased job compensation, loss of productivity, and an inability to live independently (12, 50, 51). Overall, schizophrenia is associated with higher annual costs per patient than other mental illnesses (21). Additionally, cognitive symptoms are related to lower treatment adherence, increased likelihood of hospital admission, and extended durations of hospital stays (21, 52, 53). Consequently, there is pressing need to develop universally accepted and effective strategies for managing cognitive symptoms of schizophrenia, as well as to expand the therapeutic options for this condition, including innovative pharmacological treatments.

Improving cognitive symptoms could enhance patient’s functionality and independence, potentially resulting in significant cost savings for the system.

### 4.5 Future directions

Given the significant discrepancy between the importance placed on cognitive symptoms and the limited detection and management of these symptoms, it is crucial to explore future directions that can bridge this gap and enhance clinical practices. To address the gaps identified in this study, several future directions can be considered. First, most participants emphasized the need for appropriate treatment detecting cognitive impairment is meaningful if an effective treatment exists; otherwise, its relevance diminishes. Therefore, specific treatments for cognitive impairment are need. Treatments include pharmacological interventions, cognitive remediation therapy, psychosocial interventions, and integrated strategies (22, 23) It is important to consider that there are ongoing clinical trials exploring new pharmacological treatments for cognitive impairment. For example, trials are investigating the safety and efficacy of drugs like iclepertin, d-serine, luvadaxistat and xanomeline-trospium (54), which show a broad interest in improving cognitive functions. These trials are decisive for identifying potential game-changers in the treatment of cognitive impairment associated with schizophrenia.

Secondly, as the results also showed, implementing comprehensive training programs for psychiatrists and PCPs on the assessment and management of cognitive symptoms in schizophrenia is essential. Specifically, encouraging the use of validated assessment tools like the MCCB, SCoRS, BACS, and SCIP in routine clinical practice.

Finally, clinical practice guidelines for the assessment and management of cognitive symptoms in schizophrenia are indispensable. These guidelines should incorporate both pharmacological and non-pharmacological interventions, providing a well-rounded approach to treatment. Providing clinicians with the necessary training and resources to implement these tools effectively will help improve the accuracy and consistency of cognitive assessments.

Overall, it would be valuable to replicate this study in other countries to explore potential similarities and differences. Despite these findings may be applicable to most developed European countries the generalizability of the results is significantly influenced by the healthcare organization in each country. While there is no data to support broader claims, further research in diverse settings would be valuable.

### 4.6 Study limitations

This study has several limitations that should be acknowledged. The sample was drawn from the Onekey database of IQVIA, which may introduce selection bias. Additionally, the PCPs in the study confirmed being in contact with patients experiencing cognitive symptoms related to schizophrenia. Therefore, it is possible that in the total population of PCPs, the management of these patients may be slightly different in terms of the presence and management of cognitive symptoms, as they could be more attuned to the nuances of the condition. The inherent limitations of qualitative studies should also be considered, such as social desirability bias. Furthermore, the study is limited to capturing the clinicians’ perception, without delving into relevant aspects regarding cognitive impairment in schizophrenia, such as cognitive reserve (55), social cognition (56), or the effects of medication (57).

In conclusion, the present study demonstrates the need of a multidisciplinary approach for the detection, assessment, and treatment of cognitive symptoms due to their impact on the functionality and quality of life of patients with schizophrenia and their social environment. The results show that there is some awareness of the problem among psychiatrists and the PCPs involved in the care of schizophrenia, but cognitive symptoms are not prioritized to the same degree as other aspects of the illness. This is largely attributed to the limited guidance and strategy and plans for detecting, assessing, and monitoring cognitive deficits in schizophrenia. Most importantly, there is a lack of effective therapeutic interventions and specific treatments for this condition.

## Data Availability

The raw data supporting the conclusions of this article will be made available by the authors, without undue reservation.

## Supplementary material

Supplementary Table 1 Sociodemographic characteristics of the participants in the quantitative phase.

Supplementary Table 2 Approaches and questionnaires used for the detection of cognitive symptoms associated with schizophrenia by specialty. [1] Conditional logic question: From the sample of patients that answer that “Through questionnaires/assessment scales” a list of scales and tools was presented to identify which they used.
