# Supplementary material for: Cognitive symptoms in schizophrenia: an analysis of awareness, assessment, and management practices among psychiatrists and primary care physicians
Source: Front Psychiatry. 2025 May 8;16:1567410. doi: 10.3389/fpsyt.2025.1567410 (PMC12095203; doi:10.3389/fpsyt.2025.1567410)
Supplement: Supplementary Table 1 — Sociodemographic characteristics of the participants in the quantitative phase. [file Table1.docx]

# Supplementary material

## Supplementary Table 1. Sociodemographic characteristics of the participants in the quantitative phase

| **Variables** | **Psychiatrists** | **PCPs** | **Total** |
| --- | --- | --- | --- |
| Number of interviewees, n (%) | 100 (44.4%) | 125 (55.6%) | 225 (100.0%) |
| **Gender** | | | |
| Male, n (%) | 53 (53.0%) | 60 (48.0%) | 113 (50.2%) |
| Female, n (%) | 47 (47.0%) | 65 (52.0%) | 112 (49.8%) |
| **Centre** | | | |
| Primary Care Practice, n (%) | - | 125 (100.0%) | 125 (100.0%) |
| Mental Health Centre, n (%) | 34 (34.0%) | - | 34 (34.0%) |
| Private hospital, n (%) | 15 (15.0%) | - | 15 (15.0%) |
| Public hospital, n (%) | 51 (51.0%) | - | 51 (51.0%) |
| **Geographical region** | | | |
| Andalucía, n (%) | 17 (17.0%) | 23 (18.4%) | 40 (17.8%) |
| Aragón, n (%) | 1 (1.0%) | 5 (4.0%) | 6 (2.7%) |
| Asturias, n (%) | 5 (5.0%) | 4 (3.2%) | 9 (4.0%) |
| Cantabria, n (%) | 1 (1.0%) | 3 (2.4%) | 4 (1.8%) |
| Castilla-La Mancha, n (%) | 4 (4.0%) | 5 (4.0%) | 9 (4.0%) |
| Castilla y León, n (%) | 5 (5.0%) | 10 (8.0%) | 15 (6.7%) |
| Cataluña, n (%) | 19 (19.0%) | 18 (14.4%) | 37 (16. 4%) |
| Extremadura, n (%) | 3 (3.0%) | 3 (2.4%) | 6 (2.7%) |
| Galicia, n (%) | 4 (4.0%) | 6 (4.8%) | 10 (4.4%) |
| Islas Baleares, n (%) | 2 (2.0%) | 3 (2.4%) | 5 (2.2%) |
| Islas Canarias, n (%) | 1 (1.0%) | 5 (4.0%) | 6 (2.7%) |
| La Rioja, n (%) | 0 (0.0%) | 2 (1.6%) | 2 (0. 9%) |
| Madrid, n (%) | 15 (15.0%) | 15 (12%) | 30 (13.3%) |
| Murcia, n (%) | 2 (2.0%) | 6 (4.8%) | 8 (3.6%) |
| Navarra, n (%) | 3 (3.0%) | 0 (0.0%) | 3 (1.3%) |
| País Vasco, n (%) | 6 (6.0%) | 5 (4.0%) | 11 (4.9%) |
| Comunidad Valenciana, n (%) | 12 (12.0%) | 12 (9.6%) | 24 (10.7%) |

## Supplementary Table 2. Approaches and questionnaires used for the detection of cognitive symptoms associated with schizophrenia by specialty

| **Items** | **Psychiatrists (N=100)** | **PCPs (N=125)** | **Total**  **(N=225)** |
| --- | --- | --- | --- |
| Clinical Criterion | 87 (87.0%) | 77 (61.6%) | 164 (72.9%) |
| Through questions asked to the patient | 85 (85.0%) | 98 (78.4%) | 183 (81.3%) |
| Through questionnaires/assessment scales [1] | 45 (45.0%) | 65 (52.0%) | 110 (48.9%) |
| MATRICS Consensus Cognitive Battery (MCCB) | 13 (28.9%) | 7 (10.8%) | 20 (18.2%) |
| Schizophrenia Cognitive Rating Scale (SCoRS) | 21 (46.7%) | 32 (49.2%) | 53 (48.2%) |
| EPICOG-SCH battery | 2 (4.4%) | 4 (6.2%) | 6 (5.5%) |
| Mini-mental test or other screening tests | 37 (82.2%) | 59 (90.8%) | 96 (87.3%) |
| BACS (Brief Assessment of Cognition Schizophrenia) | 17 (37.8%) | 7 (10.8%) | 24 (21.8%) |
| SCIP (Screen for Cognitive Impairment in Psychiatry) | 5 (11.1%) | 10 (15.4%) | 15 (13.6%) |
| Others | 2 (4.4%) | 1 (1.5%) | 3 (2.7%) |
| Diagnosed by another specialist | 7 (7.0%) | 28 (22.4%) | 35 (15.6%) |
| Others | 2 (2.0%) | 3 (2.4%) | 5 (2.2%) |

[1] Conditional logic question: From the sample of patients that answer that “Through questionnaires/assessment scales” a list of scales and tools was presented to identify which they used.
